# Coverage and associated factors of inactivated polio vaccine uptake among children aged 12–23 months in Sub-Saharan Africa

**DOI:** 10.1038/s41598-026-40258-3

**Published:** 2026-03-11

**Authors:** Wubet Tazeb Wondie, Alemu Birara Zemariam, Zenebe Abebe Gebreegziahber, Bruck Tesfaye Legesse, Kefyalew Taye Belete, Geberehiwot Berie Mekonnen, Gezahagn Demsu Gedefaw, Wabi Temesgen Atinafu, Beminate Lemma Seifu, Wubet Tazeb

**Affiliations:** 1 Department of Pediatrics and Child Health Nursing, College of Health Sciences and Referral Hospital, Ambo University, Ambo, Ethiopia; 2 Department of Pediatrics and Child Health Nursing, School of Nursing, College of Medicine and Health Sciences, Woldia University, Woldia, Ethiopia; 3 Department of Epidemiology and Biostatistics, School of Public Health, Asrat Woldeyes Health Science Campus, Debre Berhan University, Debre Berhan, Ethiopia; 4 Department of Pediatrics and Neonatal Nursing, School of Nursing and Midwifery, Institute of Health Science, Wollega University, Nekemet, Ethiopia; 5 Department of Public Health, College of Health Sciences and Referral Hospital, Ambo University, Ambo, Ethiopia; 6 Department of Pediatrics and Child Health Nursing, College of Health Science, Debre Tabor University, Debre Tabor, Ethiopia; 7 Department of Neonatal Health Nursing, School of Nursing, College of Medicine and Health Science, University of Gondar, Gondar, Ethiopia; 8 Department of Public Health, College of Medicine and Health Sciences, Samara University, Samara, Ethiopia

**Keywords:** Children 12–23 months, Coverage, Determinants, Inactivated polio vaccine, Sub-Saharan africa, Medical research, Health care, Disease prevention, Health services, Paediatrics, Public health

## Abstract

The Inactivated Polio Vaccine (IPV) protects against all strains of the polio virus and poses no risk of vaccine-associated paralytic poliomyelitis. However, its coverage remains below an optimal level in Sub-Saharan Africa (SSA), and instances of vaccine-derived poliovirus have been reported. While several studies explored vaccine coverage, research specifically focused on IPV in SSA remains limited. Hence, this study aimed to assess the coverage and determinants of IPV uptake among children 12–23 months of age. A secondary data analysis was conducted using data from the recent demographic and health survey in 20 SSA countries between 2016 and 2023. The study included a total weighted sample of 43,564 children aged 12–23 months. Due to the hierarchical nature of the data, multilevel logistic regression was employed to identify associated factors. Model fitness and comparison were assessed using the median odds ratio, intra-class correlation coefficient, proportional change in variance, and deviance. Adjusted Odds Ratios (AORs) with their 95% CI were computed. Variables with a P-value < 0.05 in the multivariable multilevel analysis were considered statistically significant. The pooled inactivated polio vaccine coverage was 65.01% with a 95% CI (55.44, 74.76). Maternal age 20–35 years (AOR = 1,08, 95% CI: 1.00, 1.18) and above 35 years (AOR = 1.22, 95%CI: 1.11, 1.34), maternal education at primary level (AOR = 1.30, 95% CI: 1.23,1.37), and secondary level or above (AOR = 1.87, 95% CI: 1.76, 1.99), marital status (married (AOR = 0.83, 95% CI: 0.75,0.90), and widowed/divorced (AOR = 0.85, 95% CI: 0.75, 0.95))), media exposure (AOR = 1.20, 95% CI: 1.14,1.25), antenatal visit 1–3 (AOR = 1.86, 95%CI: 1.72, 2.01) and *≥* 4 visit (AOR = 2.51, 95% CI: 2.33,2.70), postnatal care (AOR = 1.73, 95%CI: 1.65,1.82), delivery at a health facility(AOR = 1.86, 95%CI: 1.77,1.97), birth interval more than 48 months (AOR = 1.34, 95%CI: 1.24, 1.44), urban residence (AOR = 1.24, 95%CI: 1.17,1.31), high community female literacy (AOR = 1.68, 95%CI: 1.54,1.83) were statistically significant positive determinants of IPV uptake. Conversely, rich household wealth (AOR = 0.84, 95% CI: 0.79,0.89) showed an inverse association. In most SSA countries, the inactivated polio vaccine coverage among children aged 12–23 months is substantially below the WHO-recommended herd immunity threshold of 90%, as well as beneath the 2024 global coverage of 85%. To improve this, stakeholders should focus on public health interventions like investing in maternal education, promoting antenatal and postnatal care, strengthening health service delivery, and raising community awareness through social media. Additionally, vaccination programs should target underserved areas and include mobile vaccination services.

## Introduction

Poliomyelitis is a highly contagious viral infectious disease caused by poliovirus, primarily affecting children under five years of age^1,2^. The virus has three types (Poliovirus 1, Poliovirus 2, and Poliovirus 3)^2^. However, wild poliovirus type 2 was declared eradicated in 2015 and type 3 in 2019, leaving only wild type 1 in circulation. These viruses are dangerous, attacking the nervous system and potentially causing irreversible paralysis, disability, and death^3^. However, it can be prevented with vaccines, which are included in the national vaccination schedule^4^. Since Polio is an incurable disease, vaccination is essential^3^. Currently, two types of Polio vaccines are used: The oral polio vaccine (OPV) and the inactivated polio vaccine (IPV). Oral polio vaccine is highly effective against poliovirus, but carries a rare risk of vaccine-associated paralytic poliomyelitis (VAPP) at a rate of approximately 1 case per 2.4–2.7 million doses administered overall (higher after the first dose). In contrast, the inactivated poliovirus vaccine (IPV) given as an injection protects against all wild-type poliovirus (WPV) strains, without any risk of vaccine-associated paralytic poliomyelitis (VAPP)^5,6^. Circulating vaccine-derived poliovirus (cVDPV) can emerge when OPV-related strains circulate and revert in under-immunized populations with low population immunity, rather than solely as a direct result of OPV use^6^. Ninety-nine percent (99%) of children who get the recommended doses of the IPV vaccine will be protected against the disease^7^. IPV is often combined with OPV to boost immunity against all types of polioviruses^8^. Since 2015, the World Health Organization (WHO) has recommended the inclusion of IPV in routine immunization schedules, and many Sub-Saharan African countries have gradually incorporated IPV into their vaccination programs^1,2^. However, disparities in coverage remain across the region. According to the WHO reports, in 2024, the global coverage of IPV was 85%^9^, with the highest coverage of 95%% in the European region, and the 93% in South-East Asia^10^, 81% in Gavi-supported countries^11^. According to the Global Polio Eradication Initiative (GPEI), in 2023, routine immunization coverage in low-income and fragile countries remained below 75%, which is insufficient to achieve community immunity. Furthermore, 85% of children affected by polio are from these countries^12^. In Sub-Saharan African countries, routine immunization coverage remains below the optimal level, with significant variation across countries and regions^13^. According to a previous study conducted in SSA, the median coverage of IPV was 73% up to the year 2014^14^, and in South and Southeast Asian countries it was 85%^14^. To end polio, the Polio Eradication and Endgame Plan 2013–2016 was developed, which included the introduction of IPV^15^. However, despite these efforts, the WHO reports outbreaks of circulating vaccine-derived poliovirus (cVDPV) in four WHO regions: Africa, Eastern Mediterranean, South-east Asian, and Western Pacific Regions^16^. In Africa, wild poliovirus transmission has been stopped, but cVDPV remains a major public health threat due to insufficient vaccination coverage^17^.

Prior studies have identified several factors influencing the uptake of IPV, including residence^18,19^, maternal education^20^, distance to health facilities, delivery at health facilities^13,21^, fathers’ educational status^22^, female household head^21^, and maternal employment status^20^. Additional factors encompass community media exposure^23,24^, antenatal care, postnatal care (PNC) utilization^13,21^, media exposure^13,21^, family wealth index, birth interval^21^, community poverty^21^, and maternal age, specifically between 15 and 24 years^20^. To boost IPV coverage, various efforts have been undertaken, for instance, in 2021, the WHO recommended that all countries introduce at least two doses of IPV in their EPI Schedule. However, currently, only 55 countries have introduced these two doses^25^.

Despite the critical importance of the IPV in the fight against Polio, studies examining its coverage and associated factors in various sub-Saharan African countries remain limited^23^. Notably, few studies have assessed IPV uptake coverage and its determinants among children aged 12–23 months using recent pooled demographic and Health Surveys (DHS) data. Evaluating IPV coverage and its associated factors in Sub-Saharan Africa is essential to understand disparities between countries. Additionally, gaining insights into individual and community-level factors affecting IPV, and develop targeted strategies to improve vaccination rates and advance efforts toward polio eradication^26^. The study also had sufficient statistical power to detect the true effect of the variables due to the large sample size and number of clusters in the DHS data, which allowed precise estimation of effects and between-cluster variance in the multilevel logistic regression model. Therefore, the present study aimed to assess the coverage of IPV uptake and its determinants in 20 Sub-Saharan African countries using the latest DHS data.

## Methods

### Data source and sampling procedures

The current study was conducted based on the most recent Demographic and Health Survey (DHS) data of 20 Sub-Saharan African countries. The study includes DHS data collected from 2016 to 2023. Survey data released before 2016 were excluded, because this might not represent the country’s current inactivated polio vaccination coverage status. The data sets of Burkina Faso, Burundi, Cote d’Ivoire, Ethiopia, Gabon, Ghana, Gambia, Guinea, Kenya, Liberia, Madagascar, Mali, Mauritania, Mozambique, Nigeria, Rwanda, Sierra Leone, Senegal, Tanzania, and Uganda were included.

The DHS data is a nationally representative survey conducted every five years in over 90 low and middle-income countries on basic health indicators such as family planning services, mortality and morbidity, HIV/AIDS, fertility, maternal and child health, nutrition, and self-reported behaviors among adults^27^. Each country’s survey consists of different datasets, including men, women, children, birth, and household datasets. For the present study, we used the kids’ record dataset (KR file). The data set used in this study was downloaded in STATA format from the DHS website, which is available from https://dhsprogram.com/data/dataset_admin/index.cfm. The datasets of the 20 SSA countries were appended together to investigate the Inactivated Polio vaccine immunization coverage and associated factors among children aged 12–23 months in SSA. The DHS data are stratified by geographic regions and within each region by urban and rural areas.

In this study, we utilized publicly available data from the Demographic and Health Surveys (DHS) Program. The DHS surveys are conducted following standardized protocols and ethical guidelines to protect respondents’ privacy and ensure data quality. All analyses were performed in compliance with DHS data use policies and institutional ethical standards. No individual-level identifiers were accessed, and confidentiality was strictly maintained throughout the study.

### Populations of the study

All children aged 12–23 months and their mothers in Sub-Saharan Africa were the target population of this study, while all alive children aged 12–23 months and their mothers in the selected enumeration area within the SSA countries where the data were collected were the study population. In this study, a total weighted sample of 43,564 children aged 12–23 months were included.

### Variables of the study

#### Dependent variable

The primary outcome variable of the study was the inactivated polio vaccination status of children aged 12–23 months. This outcome variable was dichotomized as “yes” for those children who received IPV, and “no” for those children who failed to take the recommended IPV immunization. The WHO recommends the intake of inactivated polio vaccine at 14 weeks of age alongside oral polio vaccine 3 (OPV3) to prevent all strains of poliovirus and vaccine-derived poliovirus. The DHS program determined the children’s IPV vaccination status from two sources these are child vaccination record cards and the mother/caretaker’s verbal reports of children’s immunization status.

#### Independent variables

Due to the hierarchical nature of the DHS data, independent variables were considered at the individual level and community level.

##### *The individual–level variables*

The individual-level variables include socio-demographic characteristics (Sex of the child, age of the child in months, mother’s age, maternal education, household wealth status, mothers’/caregiver marital status, sex of household, media exposure), and Child and maternal-related factors (ANC visit, PNC visit, mode of delivery, birth order, birth interval).

**Community-level factors**: The community-level variables were community media exposure, community women’s literacy, community poverty, the Sub-Saharan Africa region, and place of residence. These community-level factors were generated by integrating individual-level factors with the cluster number because, except for residency and country in DHS, all variables were collected at the individual level. The variables were dichotomized as low and high using the median value because the data were not normally distributed. We generated the variable “media exposure” using three components: watching television, reading newspapers or magazines, and listening to the radio. Parents/caregivers who were involved in any of these activities–listening to the radio or watching television or reading newspapers/magazines at least once a week were classified as having media exposure, coded as “Yes”. Those who did not meet this criterion were classified as not having media exposure and coded as “No”. In this study, the variables were categorized and recorded based on the analytical framework used by the DHS.

### Data management and analysis

The variables of this study were obtained from the Kids Record (KR) file, and the data were kept, cleaned, recorded, and analyzed using STATA version 17 statistical software. The extracted data from 20 Sub-Saharan African countries were appended together, and the sample was weighted using sampling weight (v005), primary sampling unit, i.e., v023, and strata, i.e., v021, to draw valid inferences. The proportion of inactivated polio vaccine coverage was presented using forest plots.

The DHS data have a hierarchical structure, which breaks the independence of observations and the equal variance assumption of traditional logistic regression. Children and women are nested within a cluster (enumeration areas), where they may share similar characteristics within the cluster. This indicates the need to account for cluster variability using an advanced model. Therefore, this study employed multilevel binary logistic regression analysis to identify the determinants of the inactivated polio vaccine Uptake. To measure variation between clusters, Intra-Clusters Correlation Coefficient (ICC), Likelihood Ratio test, Proportional Change in Variance (PCV), and Median Odds Ratio (MOR) were computed. Model comparison was made based on deviance, which was computed as -2(log-likelihood ratio) since the model had a different parameter. The model with the lowest deviance was considered the best-fitted model. The Intra-Clusters Correlation Coefficient (ICC) quantifies the degree of heterogeneity in IPV uptake between clusters, representing the proportion of total observed differences in IPV immunization attributable to cluster variations^28^. It was calculated using the formula: ICC=$\:\frac{\mathrm{V}\mathrm{C}}{\mathrm{V}\mathrm{C}+3.29}\:\times\:100\mathrm{\%}$, where VC represents cluster variance.

The Median Odds Ratio (MOR) was used to assess the variability in the odds of IPV uptake between clusters. It is defined as the median odds ratio when comparing clusters with higher odds of IPV immunization to those with lower odds, based on the random selection of two clusters or countries. The MOR was calculated using the formula: MOR= $\:{\mathrm{e}}^{0.95\surd\:\mathrm{v}\mathrm{c}}$.

The Proportional Change in Variance (PCV) was used to evaluate the total variance in IPV immunization explained by individual and community factors in the final model compared to the null model. The PCV was calculated using the formula.

PCV= $\:\frac{\mathrm{V}\mathrm{a}\mathrm{r}\:\mathrm{n}\mathrm{u}\mathrm{l}\mathrm{l}-\mathrm{V}\mathrm{a}\mathrm{r}\:\mathrm{f}\mathrm{u}\mathrm{l}\mathrm{l}}{\mathrm{V}\mathrm{a}\mathrm{r}\:\mathrm{n}\mathrm{u}\mathrm{l}\mathrm{l}}\times\:100\mathrm{\%}$, where Var null = variance of the null model, and Var full = variance of the cluster for the respective model.

Four models of multilevel logistic regression were fitted. These were the null model, model I, model II, and model III. Null model- a model without explanatory variables was used to determine the variability of IPV immunization among clusters. In Model I, the association of individual-level factors with the outcome variable was evaluated. In model II, the association of community-level factors with IPV immunization was evaluated. The final model (model III) was fitted to assess the association of both individual and community variables with IPV immunization.

Both bi-variable and multivariable multilevel logistic regression analyses were fitted. Both individual and community-level variables with a P-value < 0.25 in the bi-variable analysis were fitted in the multivariable model. To declare the statistically significant associated factors of IPV, the adjusted odds ratio (AOR) with a 95% Confidence Interval (CI), and P-value < 0.05 in the final model were reported. The variance inflation factor was used to check the multicollinearity by doing the pseudo-linear regression analysis.

## Results

### Socio-demographic and economic characteristics of the study participants

A total weighted sample of 43,564 children aged 12–23 months from 20 Sub-Saharan African Countries were included. Of these 22,247 (51.07%) were males, and more than two-thirds of children resided in rural areas. By country, Nigeria contributed the largest proportion (13.91%), followed by Kenya (8.45%), Uganda (6.71%), and the lowest proportion was from Ethiopia (2.31%). The majority of mothers were in the age group 20–34 years, and 38.33% had no formal education. 66.83% of mothers/caregivers had media exposure, and 87.52% were married women. The majority of community women have low literacy levels, and 67.90% of mothers have a high poverty level (Table 1).

**Table 1:** Socio-demographic characteristics of the study participants.

Variables	Categories	Frequency	Percent
Country	Burkina Faso	2,313	5.31
	Burundi	2,596	5.96
	Cote divore	1,920	4.41
	Ethiopia	1,008	2.31
	Gabon	1,271	2.91
	Ghana	1,946	4.47
	Gambia	1,582	3.63
	Guinea	1,408	3.23
	Kenya	3,679	8.45
	Liberia	1,063	2.44
	Madagascar	2,345	5.38
	Mali	1,946	4.47
	Mauritania	2,119	4.86
	Mozambique	1,727	3.96
	Nigeria	6,059	13.91
	Rwanda	1,572	3.61
	Sierra Leone	1,861	4.27
	Senegal	2,057	4.72
	Tanzania	2,143	4.92
	Uganda	2,922	6.71
Sex of the child’s head.	Male	22,247	51.07
	Female	21,317	48.93
Age of the child in months	12–15	15,822	36.32
	16–19	14,824	34.03
	20–23	12,918	29.65
Mothers age	15–19	3,379	7.76
	20–34	30,741	70.57
	*≥* 35	9,444	21.68
Residence	Rural	29,777	68.35
	Urban	13,787	31.65
Maternal education	No formal education	16,935	38.87
	Primary Secondary and above	13,341 13,288	30.62 30.50
Household wealth status	Poor	20,873	47.91
	Middle	8,600	19.74
	Rich	14,091	32.35
Mothers/caregiver marital status	Single	2,954	6.78
	Married	38,126	87.52
	Widowed/divorced/separated	2,484	5.70
Sex of head of the household	Male	34,671	79.59
	Sex	8,893	20.41
Media exposure	No	14,449	33.17
	Yes	29,115	66.83
Community poverty level	Low	13,985	32.10
	High	29,579	67.90
Community women’s literacy level	Low	37,859	86.90
	High	5,705	13.10
Community media exposure	Low	10,104	23.19
	High	33,460	76.81

### Child and maternal-related characteristics

Of the total children, 72.39% were born in health facilities, and 62.41% of mothers received antenatal care at nearby health facilities. The majority of children were born vaginally, and 72.08% of mother-child pairs did not have a postnatal check-up (Table 2).

**Table 2:** Child and maternal-related characteristics of children 12–23 months of age in Sub-Saharan African countries.

Variables	Response	Weighted frequency	Percent
Place of delivery	Home Health facility	12,027 31,537	27.61 72.39
ANC visit	No	4,486	10.30
	1–3	11,889	27.29
	*≥* 4	27,189	62.41
PNC visit	No	31,399	72.08
	Yes	12,165	27.92
Mode of delivery	Caesarean delivery	3,056	7.01
	Vaginal delivery	40,508	92.99
Birth interval	< 23 months	5,630	12.92
	24–48 months	28,373	65.13
	> 48 months	9,561	21.95

### Coverage of inactivated polio vaccine

**Fig. 1:**
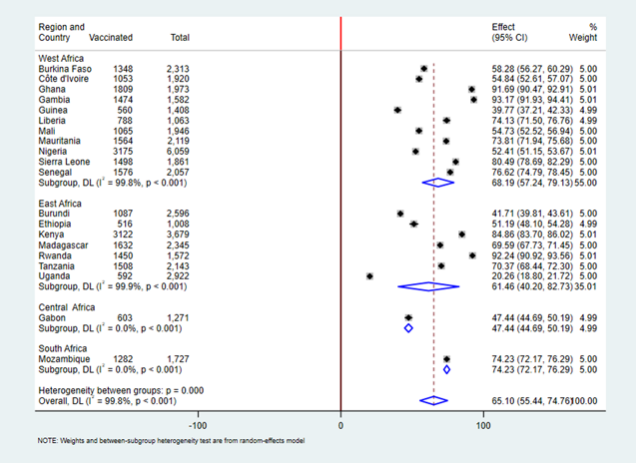
Forest plot showing the pooled inactivated polio vaccine immunization coverage among children aged 12–23 months in Sub-Saharan African Countries.

The pooled proportion of inactivated polio vaccine coverage among children 12–23 months of age in SSA countries was 65.10% (95% CI: 55.44, 74.76). The coverage has regional disparities, in West Africa 68.19 (95% CI: 57.24, 79.13), East Africa 61.46 (95%CI: 40.20, 82.73), Central Africa 47.44 (95%CI: 44.69, 50.19), and Southern Africa 74.23 (72.17, 76.9). The Coverage of IPV uptake also varied from country to country. The highest coverage rate was found in Gambia, 93.17% (95% CI: 91.93, 94.41), and the lowest coverage was observed in Uganda 20.26% (95% CI:18.80, 21.72). Additionally, the IPV vaccination coverage also varied by place of residency, which was 71.69 in urban areas % coverage and 59.84% in rural areas (Fig. 1).

### Measures of variation (Random effect) and model fit statistics

The ICC value in the null model was 0.064 (95% CI 0.523, 0.08). This value indicates that the variation between clusters (countries) was responsible for around 6.4% of the overall variability of inactivated polio vaccine immunization. Additionally, the median odds ratio was 1.96 (95%CI: 1.88, 2.03), which means if we randomly select a child from two separate clusters (countries), the odds of receiving IPV are 1.96 times higher in one cluster (country) compared to another. This suggests a strong association between the cluster and the likelihood of vaccination (Table 3).

**Table 3:** Random effect and model fitness statistics of inactivated polio vaccine immunization in Sub-Saharan African countries.

Parameters	Null model	Model 1	Model 2	Model 3
Random effect				
Variance	0.22 (0.18 0.27)	0.10 (0.08, 0.12)	0.17 ( 0.1, 0.20)	(0.10 (0.08 0.13)
ICC	0.064 (0.05 0.08)	0.03 (0.023 0.036)	(0.05 (0.04, 0.06)	0.03 (0.02 0.04)
MOR	1.96 (1.88, 2.04)	1.56 (1.48,165)	1.79 (1.90,)	1.56 (1.49,1.67)
PCV	Reference	0.55	0.22	0.55
**Model fit statistics**				
LL	-28354.85	-26472.78	-27722.01	-25968.61
LR test	chibar2(01) = 424.07 P-value 0.00	chibar2(01) = 175.97 P-value = 0.00	chibar2(01) = 369.97 P-value = 0.00	chibar2(01) = 180.13 P-value = 0.00
Deviance	56,709.70	52,945.56	55,444.01	51,937.21

ICC= Intraclass Correlation Coefficient, LL = Log likelihood, LR test=Likelihood ratio test, MOR= Median odds Ratio, PCV= Proportional Change in Variance.

### Associated factors of inactivated polio vaccine coverage among children aged 12–23 months

In the final multivariable multilevel logistic regression analysis, maternal age, maternal education, rich household wealth status, marital status, media exposure, ANC, delivery in a health facility, PNC, birth interval above 48 months, urban residency, high community literacy, and Sub-Saharan Africa regions were significantly associated factors.

Children from mothers aged 20 to 34 years and *≥* 35 years had 1.08 times (AOR = 1,08, 95% CI: 1.00, 1.18) and 1.22 times (AOR = 1.22, 95% CI: 1.11, 1.34) higher odds of receiving IPV compared to children from mothers aged 15–19 years, respectively. Similarly, mothers attending primary school and secondary school or above were 1.30 times (AOR = 1.30 (95% CI: 1.23,1.37) and two times (AOR = 1.87(95% CI: 1.76, 1.99) at higher odds of vaccinating their children with the IPV compared with mothers with no formal education.

In the current study, children from rich households had 16% lower odds of receiving the IPV compared to children from poor households (AOR = 0.84, 95% CI: 0.79,0.89). Similarly, married women and divorced/separated women were 17% and 15% less likely to vaccinate their children with inactivated polio vaccine compared with single mothers (AOR = 0.83, 95% CI: 0.75,0.90), and (AOR = 0.85, 95% CI: 0.75, 0.95) respectively. Mothers who had media exposure were 1.20 times more likely (AOR = 1.20, 95% CI: 1.14,1.25) to vaccinate their children with the inactivated polio vaccine compared with mothers who had no media exposure. Children born to a mother who attended 1–3 ANC were nearly two times (1.86, 95%CI: 1.72,2.01) and those whose mothers attended four or more ANC visits were two and a half times more likely (AOR = 2.51, 95% CI: 2.33,2.70) to receive the inactivated polio vaccine compared to children born to a mothers who did not attend antenatal care. Children born at health facilities were two times more likely to receive inactivated polio vaccine compared with children born at home (AOR = 1.86, 95%CI: 1.77,1.97). Mothers who had PNC had 1.73 times higher odds of vaccinating their children with inactivated polio vaccine compared to children from mothers who did not receive PNC (AOR = 1.73, 95%CI: 1.65,1.82). Children born at a preceding birth interval above 48 months were 1.34 times (AOR = 1.34, 95%CI: 1.24, 1.44) more likely to be vaccinated IPV compared to children born less than 24 months of the preceding birth.

Children from urban areas were 1.24 times more likely to receive inactivated polio vaccine compared with rural area children (AOR = 1.24, 95%CI: 1.17,1.31). Likewise, children from high community women literacy were 1.68 times (AOR = 1.68, 95%CI: 1.54,1.83) more likely to receive IPV than their counterparts. Additionally, children from Eastern Africa had 3.67 times higher odds (AOR = 3.67, 95% CI: 3.22,4.19), Western Africa 4.86 times higher odds (AOR: 4.86, 95% CI: 4.27,5.53), and Southern Africa 8.41 times higher odds (AOR = 8.41=, 95%CI: 7.08, 9.99) of receiving IPV compared to children from Central Africa (Table 4).

**Table 4:** Individuals and community-level factors associated with inactivated polio vaccine uptake (IPV) among children aged 12–23 months in Sub-Saharan African countries.

Variables	Categories	Model I (AOR 95% CI)	Model II (AOR 95% CI)	Model III (AOR 95% CI)
Maternal age	15–19	1		**1**
	20–34	1.04 (0.96, 1.13)		**1.08 (1.00**, 1.18)**
	> 35	1.15 (1.05,1.26)		**1.22 (1.11**, 1.34)**
Maternal education	No formal education	1		**1**
	Primary	1.21 (1.15,1.27)		**1.30 (1.23**,1.37)**
	Secondary and above	1.75 (1.64, 1.85)		**1.87 (1.76**, 1.99)**
Household Wealth Status	Poor	1		**1**
	Middle	1.04 (0.99,1.11)		0.95 (0.89,1.01)
	Rich	1.03 (0.98,1.09)		**0.84 (0.79**, 0.89)**
Marital status	Single	**1**		**1**
	Married	0.94 (0.86,1.03)		**0.83 (0.75**, 0.90)**
	Widowed/divorced/ Separated	0.99 (0.83,1.05)		**0.85 (0.75**, 0.95)**
Media exposure	No	1		**1**
	Yes	1.18 (1.12, 1.23)		**1.20 (1.14**, 1.25)**
ANC	No	1		**1**
	1–3	1.73 (1.61,1.87)		**1.86 (1.72**, 2.01)**
	4 or above	2.38 (2.21,2.56)		**2.51 (2.33**, 2.70)**
Place of delivery	Home Health facility	1 1.81(1.72,1.90)		**1** **1.86 (1.77**, 1.97)**
PNC	No Yes	1 1.67 (1.59, 1.75)		**1** **1.73 (1.65**, 1.82)**
Birth interval	< 23 months 24–48 months > 48 months	1 1.10 (1.04, 1.17) 1.39 (1.29, 1.50)		**1** 1.05 (0.98, 1.12) **1.34 (1.24**, 1.44)**
Residence	Rural		**1**	**1**
	Urban		1.71(1.63,1.79)	**1.24 (1.17**, 1.31)**
Community-women literacy	Low		**1**	**1**
	High		2.23 (2.04.2.45)	**1.68 (1.54**, 1.83)**
Community poverty level	Low		0.97 (0.90,1.05)	0.96 (0.90, 1.03)
	High		1	**1**
Sub-Saharan African Regions	Central Africa		1	**1**
	Eastern Africa		2.14 (1.90,2.41)	**3.67(3.22**, 4.19)**
	Western Africa		2.51 (2.23,2.83)	**4.86 (4.27**, 5.53)**
	Southern Africa		4.14 (3.53,4.85)	**8.41 (7.08**, 9.99)**

N.B.: ** p-value < 0.001, AOR: Adjusted Odds Ratio, CI: Confidence Interval, ANC: Antenatal Care, PNC: Post Natal Care.

## Discussion

This study evaluated the pooled coverage and determinants of inactivated polio vaccine (IPV) uptake among children 12–23 months of age in Sub-Saharan Africa, using the most recent Demographic and Health Survey (DHS) data. The pooled coverage of IPV uptake among children in Sub-Saharan Africa was 65.10% (95% CI: 55.44, 74.76), with considerable variation between countries. This coverage rate was lower than the 2024 WHO global estimate of 85%^9^, the European region of 95%^29^, Gavi-supported countries 81%^11^, and the Southeast Asian countries 93%^10^. This low coverage indicates insufficient population immunity to interrupt poliovirus transmission effectively, and herd immunity levels have not reached in the region. The lower coverage in Sub-Saharan Africa is likely due to underdeveloped healthcare systems, limited infrastructure, a shortage of healthcare workers, and poor community literacy rates in Sub-Saharan countries, which impedes effective IPV delivery^30,31^. Another reason may be the financial challenges faced by SSA countries, which restrict the provision of widespread immunization, especially in countries not participating in initiatives like Gavi. Many SSA countries rely on international donors, which creates inconsistencies in IPV delivery^32^. Additionally, cultural beliefs, political instability, and weak routine immunization systems further contribute to the low coverage^33,34^.

On the other hand, the finding of this study aligns with a previous study conducted in Sub-Saharan Africa, which reported a coverage rate of 73%^14^. This consistency might be attributed to the reliance on international funding for vaccination programs in SSA countries, fluctuations in such funding, or the lack of substantial increases over time, which may have limited improvement in IPV coverage. Another reason could be persistent challenges, such as inadequate healthcare infrastructure, widespread low socioeconomic status, limited maternal education, and logistical issues, particularly in maintaining a cold chain system. Additionally, similarities in methodology, including comparable sampling procedures and overall immunization trends in Sub-Saharan Africa, may have contributed to these consistent findings^35^.

Our finding from the multilevel multivariable logistic regression analysis also revealed that the following factors were associated with IPV immunization in the Sub-Saharan Africa region: maternal age 20–35 years and above 35 years, maternal education at primary level, and secondary level or above, wealthier household, marital status (married, and previously married (widowed/divorced/separated)), Parental media exposure, having ANC, delivery at a health facility, postnatal care, birth interval more than 48 months, urban residence, high level of literacy among women in the community.

In this study, we found that children from mothers aged 20–35 and above 35 years had higher odds of IPV immunization compared with children from mothers/caregivers aged below 20 years. This finding was consistent with studies conducted in East Africa^21^and Nigeria^36^. This might be due to the greater child-rearing experience of mothers aged 20–34 and above 35 years compared with those under 20 years. These groups of mothers are knowledgeable and familiar with the importance of immunization, immunization schedules, and healthcare practices, including IPV, which increases the likelihood of children receiving IPV^37,38^. The other reason could be that mothers in the age group have economic stability, good social networks, authority, and decision-making power in the family and the community compared with younger mothers. These factors allow these mothers to proactively seek out IPV immunizations^39^.

Similarly, maternal education, primary, secondary, or higher, and high community literacy among women were significant determinants of IPV immunization. This finding was supported by a study conducted in East Africa^21^, Indonesia^20^, Nigeria^36^, Senegal^40^, and Sub-Saharan Africa^13^. The possible reason for this is that educated mothers have good knowledge, perception, and trust in the importance of immunization, including IPV. As a result, they are more likely to vaccinate their children compared with illiterate mothers^38^. Furthermore, educated mothers typically engage more actively with healthcare systems, making them more likely to schedule IPV and other health services and attend health appointments consistently^41,42^. In addition to the factors already discussed, educated mothers possess the literacy skills necessary to independently read and comprehend Information, Education, and Communication (IEC) materials on immunization, thereby enhancing vaccine confidence and adherence.

Children whose mothers attend antenatal care (ANC) have higher odds of being vaccinated compared to those whose mothers do not attend ANC. A consistent finding was reported in Senegal^40^, Nepal^43^, East Africa^21^, Ethiopia^19^, and SSA^13^. The reason for this is that during antenatal care visits, healthcare providers educate mothers about vaccinations, address any misperceptions, and concerns they may have, which helps to improve their knowledge and attitude towards IPV, ultimately leading to better IPV uptake^44^. Another reason is that ANC helps mothers navigate the healthcare system and enhances their access to maternal and child health services. It fosters trust in healthcare providers, making ANC a critical point for inactivated polio vaccine (IPV) immunization^45^. Similarly, Postnatal Care (PNC) is a key determinant of IPV immunization among children aged 12–23 months. A consistent finding was reported in Ethiopia^46^, East Africa^21^, and Sub-Saharan Africa^13^. This might be because PNC visits enable healthcare providers to educate mothers about the importance of immunization, remind them of vaccination schedules, offer counseling, and track their children’s vaccination status^47^. Additionally, mothers who attend PNC are more likely to access maternal and child health services^45,46^.

Contrary to the expectations and prior studies from Ethiopia^46^, Nepal^43^, East Africa^21^, and Sub-Saharan Africa^13^. which consistently report higher IPV uptake among wealthier households, our study revealed an inverse association between the richest household wealth quantile and IPV uptake (AOR = 0.84, 95% CI: 0.79–0.89). This finding may reflect vaccine hesitancy among wealthier urban households, possibly driven by higher education level or preferences for competing private healthcare options, whereas targeted outreach campaigns may improve IPV access in poorer rural area^48^. Reversal causality is also possible, where IPV uptake proxies broader health-seeking behaviors more prevalent among lower wealth groups benefiting from subsidized services. Further studies should explore these wealth gradients through stratified analyses and qualitative assessments of service delivery barriers.

Children from married or previously married (widowed/divorced/separated) parents were more likely to be immunized with IPV compared to those of single mothers. Similar finding was reported from studies conducted in Gahanna^49 ^and Japan^50^. Married or previously married women have better financial stability, allowing them to afford healthcare-related expenses such as transportation, time off work, and other indirect costs associated with accessing immunization services^51^. Additionally, married women are more integrated into community health programs and often receive more social support from partners, extended family, and community networks. This support can enable these mothers to prioritize healthcare for their children when they have both physical and emotional demands for assistance^52^.

Consistent with studies conducted in Somalia^53^, Senegal^40^, Nepal^43^, Nigeria^54^, and Sub-Saharan Africa^13^, in this study, delivery at health facilities was a significantly associated factor of IPV immunization in children. This association might be attributed to the early introduction of immunization schedules by healthcare providers, accessibility of information and education about the importance of vaccination, and the risks of missing doses immediately after delivery^47,55^. Additionally, delivering in a health facility establishes a direct link with the health care system, reducing traditional barriers and encouraging timely IPV vaccination^47^.

Parents’ media exposure was significantly associated with increased odds of inactivated polio vaccination, this finding was consistent with studies conducted in Uganda^23^, Ethiopia^24^, East Africa^21^, and Sub-Saharan Africa^13^. Social media plays a great role in sharing and transferring information about the benefits of the IPV and the consequences of missing IPV, which raises parents’ awareness and motivates parents to prioritize IPV immunization for their children^56^. Additionally, the media debunk misinformation and myths, fostering trust in vaccine safety and efficacy. This helps to create a positive social norm around IPV and makes parents feel more comfortable and confident^57^. Furthermore, media helps to leverage targeted interventions and campaigns to reach high-risk or underserved communities, ensuring they are receiving relevant information^58^. Women who gave birth after a preceding birth of 48 months or more were more likely to vaccinate their children with the IPV compared to women who gave birth within 24 months of the preceding birth. This finding is supported by studies conducted in East Africa^21^, and Bangladesh^59^. Women with longer birth intervals may have time and resources to dedicate to each child and pay good attention to the health care needs of children^60,61^. Additionally, shorter birth intervals are often associated with adverse health outcomes for both mother and children which affects their access to IPV^62^.

As supported by studies conducted in Nigeria^63^, Ethiopia^64^, Sub-Saharan Africa^65^, and India^66^, Children from urban residents had a higher likelihood of being vaccinated with the inactivated polio vaccine compared with children from rural residents. Urban areas generally have more developed healthcare infrastructure, with well-stocked facilities and a larger, more stable workforce that is easier to access. This setup encourages regular healthcare visits, ensures vaccine availability, and supports consistent immunization services^67^. Besides, urban residents have greater access to health education resources, and vaccination campaigns are often focused on urban areas due to higher population density. This increased exposure to information and immunization efforts raises the likelihood that children in urban areas will receive the IPV^68^.

## Conclusion and recommendations

This study found that the pooled IPV coverage among children aged 12–23 months in most SSA countries is substantially below the WHO-recommended herd immunity threshold of 90%^69^, as well as beneath the 2024 global coverage of IPV 85%^9^. This low coverage indicates insufficient population immunity to interrupt poliovirus transmission effectively in the region. Maternal age 20–35 and above 35 years, maternal education primary level, and secondary level or above, marital status (married, and previously married (widowed/divorced/separated)), media exposure, antenatal and postnatal care visit, health facility delivery, birth spacing of over 48 months, urban residence, higher community literacy among women were factors that improve the uptake of IPV. Conversely, rich household wealth showed an inverse association.

This low coverage suggests that herd immunity levels are not reached in most settings, leaving communities vulnerable to poliovirus transmission and outbreak. The gap poses a significant challenge to polio eradication in the region. Hence, addressing socio-demographic and healthcare access factors is critical to reaching herd immunity levels needed for polio eradication. Hence, by considering the aforementioned factors, the stakeholders should prioritize public health interventions such as investing in maternal education, promoting ANC and PNC visits, strengthening health service delivery systems, especially in rural areas, and using media to raise community awareness. Additionally, vaccination programs should prioritize reaching underserved populations by implementing mobile vaccination services to increase IPV coverage and advance toward polio eradication goals. Further studies should investigate these inverse wealth gradients in IPV uptake comparing poor and rich household groups using stratified analyses by socioeconomic status, alongside qualitative assessments of service delivery barriers and vaccine hesitancy drivers.

### Strengths and limitations of the study

This study utilized Demographic and Health Survey (DHS) data from sub-Saharan Africa (SSA), which includes large populations across multiple countries. The DHS data is cross-sectional data, which cannot establish causal inferences between explanatory variables and outcome variables. The DHS data has detailed information about individual and community-level factors, however, it has limited data about health system-related factors. Additionally, reliance on mother/caretaker verbal reports alongside vaccination cards may introduce recall bias, potentially leading to over- or underestimation of IPV coverage, though DHS surveys standardize this approach to minimize such effects. There may also be a time lag between data collection and release, meaning the data might not reflect the most recent shifts in health service delivery.

## Data Availability

The datasets generated and analyzed for the present study are available in the DHS website (which is available from [https://dhsprogram.com/data/available-datasets.cfm](https:/dhsprogram.com/data/available-datasets.cfm) .

## Abbreviations

ANC: Antenatal care
AOR: Adjusted Odds Ratio
CI: Confidence Interval
DHS: Demographic and Health Survey
ICC: Intra-class correlation coefficient
IPV: Inactivated Polio Vaccine
LL: Log Likelihood Ratio
LLR: Log Likelihood Ratio
MOR: Median Odds Ratio
PCV: Proportional Change in Variance
PNC: Post Natal Care
SSA: Sub-Saharan Africa
VAPP: Vaccine-Associated Paralytic Poliomyelitis
WHO: World Health Organization
